# A Novel Case of a Massive Amebic Liver Abscess in the Setting of Uncontrolled Diabetes Mellitus and Concurrent Pulmonary Embolism

**DOI:** 10.7759/cureus.52533

**Published:** 2024-01-18

**Authors:** Mohamed Ismail, Menna-Allah Elaskandrany, Sahiba Singh, Natalia Chmielewska, Weizheng Wang

**Affiliations:** 1 Department of Internal Medicine, New Jersey Medical School, Rutgers University, Newark, USA; 2 Department of Internal Medicine, Lenox Hill Hospital, Manhattan, USA; 3 College of Osteopathic Medicine, Michigan State University, East Lansing, USA; 4 Department of Gastroenterology and Hepatology, New Jersey Medical School, Rutgers University, Newark, USA

**Keywords:** pulmonary embolism (pe), uncontrolled diabetes mellitus, elevated liver enzyme, right upper quadrant abdominal pain, bacterial liver abscess, amebic liver abscesses

## Abstract

Amebic liver abscesses (ALAs), one of the most common extraintestinal manifestations of invasive amebiasis, pose diagnostic challenges due to their various clinical presentations and difficulty in distinguishing them from pyogenic abscesses. This case presentation highlights the intricacy of determining the source of an unusually large liver abscess that had an even rare occurrence of a coinciding pulmonary embolus without any evidence of a deep vein thrombosis. This unusual combination underscores the challenges in identifying and managing atypical cases of ALA and emphasizes the need for more comprehensive data to enhance our understanding of such occurrences.

## Introduction

Hepatic abscesses can arise from bacterial or amebic infections and can be categorized as amebic or pyogenic [1]. They share clinical features such as fever, chills, right upper quadrant abdominal pain, jaundice, weight loss, and/or nausea and vomiting, making diagnosis challenging. However, the distinction is necessary as they differ in management and treatment options [2,3]. Amebic liver abscesses (ALAs) result from hepatocyte death via apoptosis or necrosis. Pyogenic abscesses differ from ALA, as ALA does not contain inflammatory cells. This is due to the protozoan's capability to break down neutrophils, leading to the formation of a substance with an *anchovy paste* consistency [1].

Amebiasis is the second leading cause of death from parasitic disease worldwide, with ALAs being the most common extraintestinal manifestations [3-5]. It is caused by the pathogen Entamoeba histolytica, which is endemic to Africa and Southeast Asia [6]. Its lifecycle consists of two stages: the cyst stage and the trophozoite stage. Cysts get ingested through contaminated food or water and get excysted into the trophozoite stage, which can then invade the intestinal epithelial cells that line the gastrointestinal (GI) tract and infect the liver via the hematogenous spread, causing hepatic inflammation, necrosis, and abscess formation [4,7,8]. This disease is very prevalent, with over 50 million infections and 100,000 deaths per year across the globe [6]. Risk factors for developing an ALA include male gender, a history of immigration from endemic areas, diabetes, and an age below 50 [3,8,9]. A timely and correct diagnosis is essential, as it is a progressive disease that can be fatal if left untreated [1].

While hepatic abscesses less than 5 cm can be managed by medicine alone [3,6], a larger size poses risks of additional complications such as rupture, peritonitis, inferior vena cava, and hepatic vein thrombosis. These complications increase the risk of thrombus formation by impeding blood flow, potentially leading to pulmonary embolism (PE) or ischemia [8,10,11], all of which are associated with higher mortality rates in comparison to uncomplicated ALA [1,5]. This case presents a rare situation where a patient exhibited an unusual ALA presentation, first deemed a pyogenic abscess, also complicated by a PE without any evidence of a deep vein thrombosis (DVT).

## Case presentation

A 61-year-old male patient, originally from Nigeria with a medical history of hypertension and uncontrolled diabetes mellitus, presented to the emergency department in the United States. He complained of persistent right upper quadrant abdominal pain, which radiates to his back, and he also noticed a darkening of his urine color. The patient had no recent symptoms of nausea, vomiting, or diarrhea, nor had he traveled outside the United States in the past two years.

**Figure 1:**
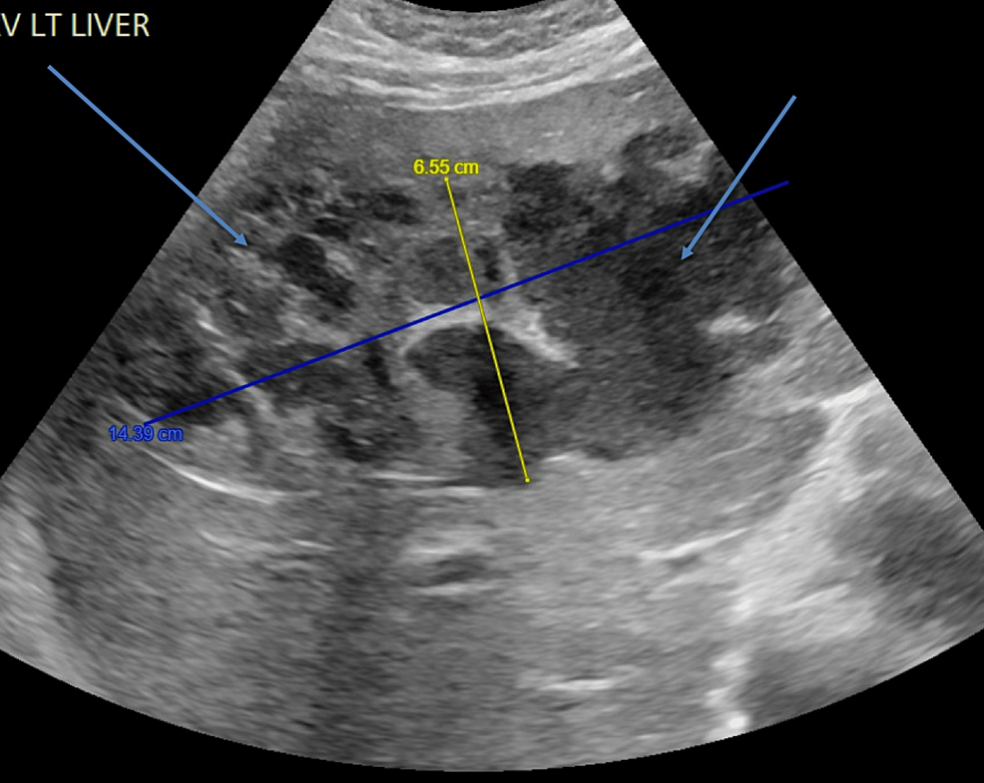
Ultrasound of the abdomen. A complex cystic mass measuring 14.39 cm x 6.55 cm in the right lobe of the liver suggests the presence of a liver abscess.

**Figure 2:**
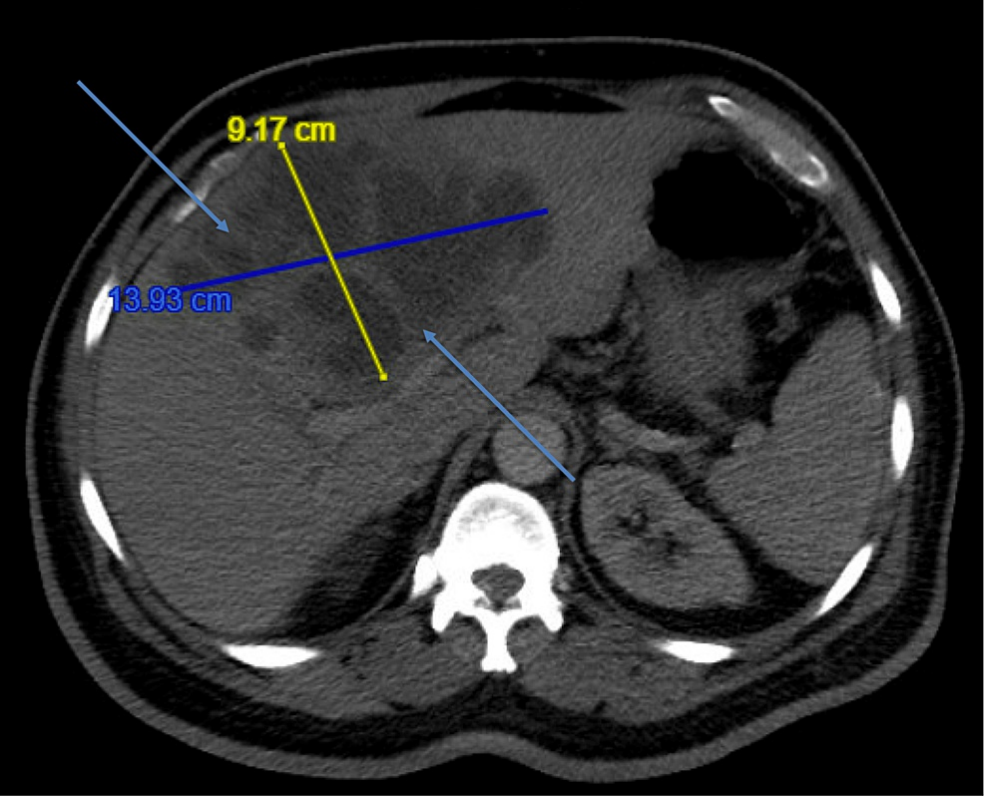
CT scan of the abdomen and pelvis with intravenous (IV) contrast. A 14 cm complex cystic lesion located within the right lobe of the liver, suggesting a high likelihood of being a giant liver abscess.

Upon examination, he had tachycardia (117 bpm), fever (100.8 °F), conjunctival icterus, and tenderness in the right upper quadrant. Laboratory tests showed elevated liver enzymes (alanine aminotransferase [ALT], aspartate aminotransferase [AST], and alkaline phosphatase), high levels of total and direct bilirubin, and anemia (hemoglobin [HGB] at 10.9) (Table 1). Diagnostic imaging included an abdominal ultrasound revealing a large complex cystic lesion in the right lobe of the liver, measuring 14.39 cm x 6.55 cm (Figure 1). A computed tomography (CT) of the abdomen and pelvis further characterized this lesion as measuring 13.93 cm x 9.17 cm, suggestive of a significant liver abscess (Figure 2).

**Table 1:** Laboratory values on admission. HbA1c, hemoglobin A1c; AST, aspartate transaminase; ALT, alanine transaminase

Lab test	Patient values	Reference range
Hemoglobin (HGB)	10.9 g/dL	14-18 g/dL
Glucose random	438 mg/dL	70-109 mg/dL
HbA1c	13.6%	4.8%-5.9%
Alkaline phosphatase	587 U/L	40-130 U/L
Bilirubin total	1.4 mg/dL	<=1.0 mg/dL
Bilirubin direct	0.8 mg/dL	<=0.3 mg/dL
AST	202 U/L	0-40 U/L
ALT	245 U/L	0-41 U/L

**Figure 3:**
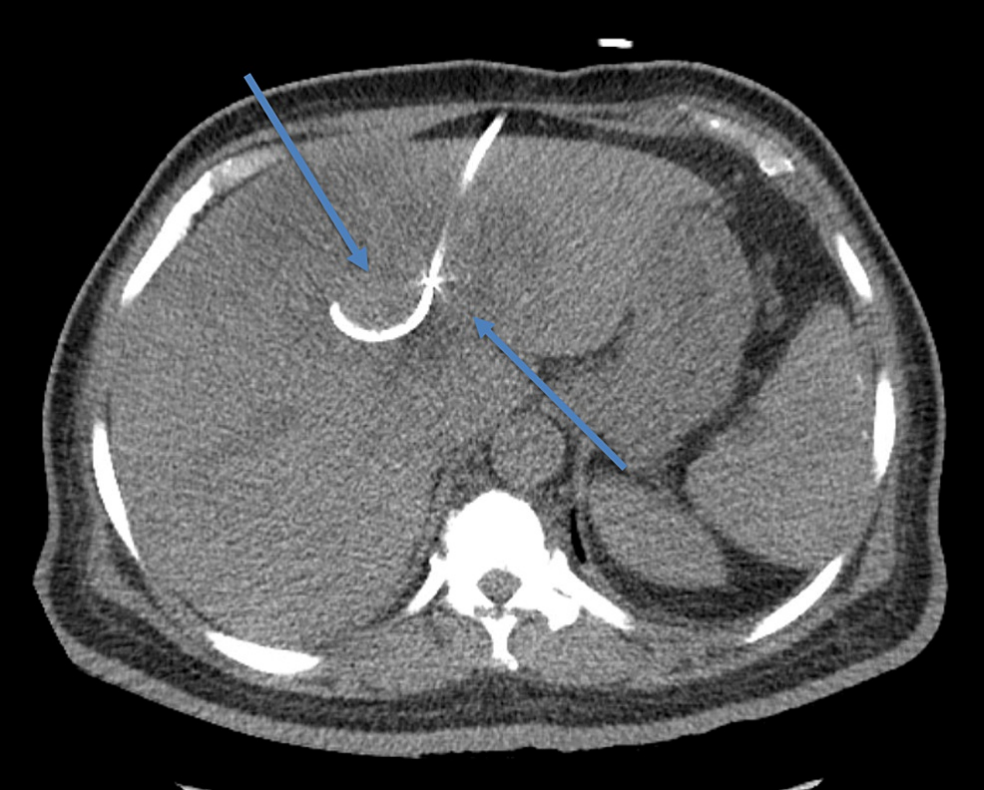
CT Abdomen with and without intravenous (IV) contrast. A large, multiloculated hypodense lesion in the liver is compatible with abscesses treated with a pigtail drainage catheter.

Initially, the patient was treated with cefepime (2 g IV every 12 hours) and vancomycin (1 g IV every 12 hours) for three days. After reviewing the CT scan results, the treatment regimen was switched to ceftriaxone (2 g IV every 24 hours) and metronidazole (500 mg IV every eight hours). Consultations were made with the General Surgery, Infectious Disease, and Interventional Radiology teams. The Interventional Radiology team conducted a CT-guided drainage of the hepatic abscess, during which a 10-French drainage catheter was inserted (Figure 3). Following the drainage, there was a significant improvement in liver function. The total bilirubin levels reduced to 0.8 mg/dL from 1.4 mg/dL, alkaline phosphatase decreased to 290 U/L from 587 U/L, and the ALT/AST levels improved to 53/49 U/L from 245/202 U/L, all observed on the fifth day of hospitalization.

During a subsequent CT scan, an incidental finding of a pulmonary embolus was detected in the right lower lobe, with no discernible origin. Liver ultrasound Doppler showed no indications of thrombosis in either the hepatic or portal veins. Consequently, the patient was instructed to take apixaban at a dose of 5 mg every 12 hours for six months to treat the unprovoked PE.

Throughout his hospitalization, the patient's blood glucose levels remained elevated (250-300s), requiring management with both long-acting and short-acting insulin. A peripherally inserted central catheter (PICC line) was also placed in the right upper arm for ongoing intravenous (IV) treatment. He was discharged from the hospital with the drainage catheter in place and scheduled for outpatient follow-ups in surgery and infectious diseases clinics.

**Figure 4:**
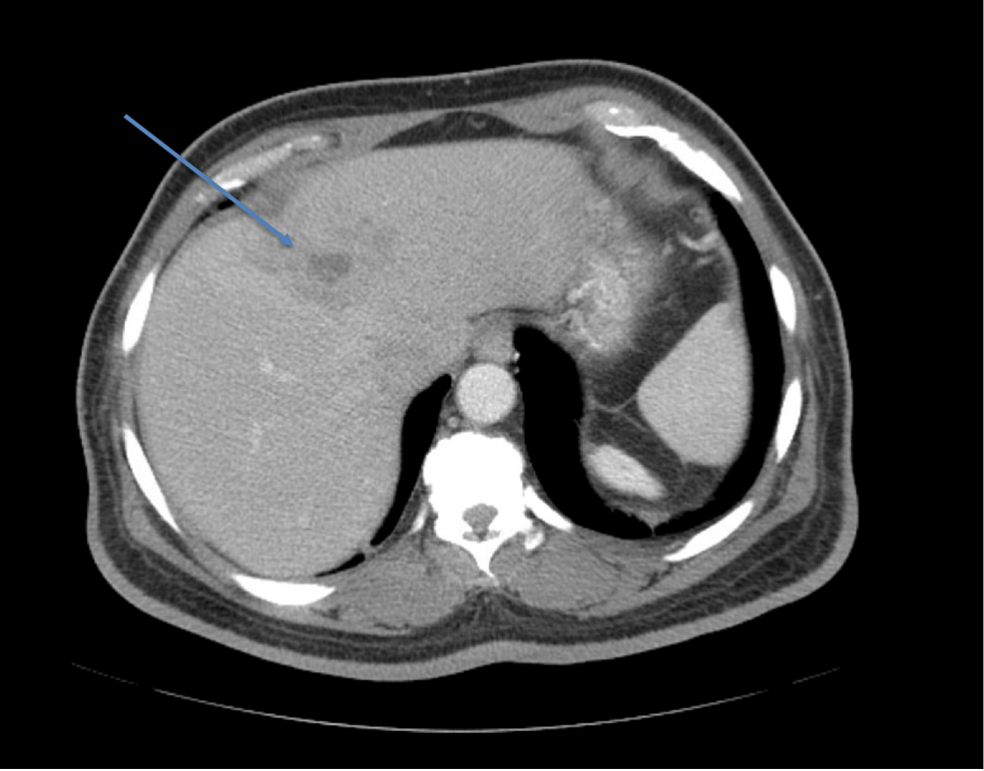
CT abdomen with intravenous (IV) contrast. Heterogeneous segment 4A with a near-complete resolution of the previous collection, and the residual discrete dominant component measuring approximately 2.1 cm x 1.6 cm x 2.0 cm.

The Infectious Disease team advised continued IV ceftriaxone (2 g IV every 24 hours) and metronidazole (500 mg IV every eight hours) through the PICC line for four weeks, with routine laboratory monitoring and scheduled outpatient CT scans. The abscess was later identified as amebic in origin after the serological testing came back positive for E. histolytica, leading to the addition of paromomycin (1,000 mg every eight hours) for seven days to the treatment regimen. A CT scan three months after treatment showed a near-complete resolution of the hepatic abscess (Figure 4).

## Discussion

ALAs of larger size and coincidental findings of pulmonary thromboembolism are uncommon in combination, with only a handful of cases documented showing their association. Moreover, the distinction between pyogenic and amebic hepatic abscesses is important for appropriate management and prevention of further complications, as delays in diagnosis have been associated with higher morbidity and mortality rates [1,5]. Meticulous history taking in patients from areas where E. histolytica is endemic can also be helpful in the correct diagnosis since the parasite can lay dormant for months, and liver abscesses can arise years after the initial infection [6].

Imaging techniques, especially ultrasonography and CT, are the mainstays of diagnosis. Ultrasonography is also helpful in differentiating between pyogenic and amebic abscesses [1,2]. It should be complemented by serological tests such as indirect hemagglutination and gel diffusion precipitation, which have high sensitivity and specificity for confirming an ALA [2]. Rapid, noninvasive tests for amebiasis have not been created yet [1].

Treatment for parasitic infection primarily involves using anti-amebic agents like metronidazole, followed by a luminal agent, to eradicate intestinal cysts and reduce the risk of recurrence [2,3,6,12]. In contrast, pyogenic abscess treatment should be individualized and involve a combination of drainage and broad-spectrum antibiotics [2].

Complications associated with ALAs are generally attributed to the inflammation and mechanical compression accompanying larger abscesses [11]. The majority of ALAs occur in the posterior surface of the right lower lobe. If they become large enough, they can compress surrounding vasculature, such as the inferior vena cava and hepatic veins, impeding blood flow and portal circulation. This creates a risk for thrombus formation, which could potentiate a pulmonary embolus [10].

## Conclusions

In our case, the patient’s 14 cm lesion was large enough to increase the risk for PE by creating a slower portal circulation; however, it remains uncertain if the PE was a result of the ALA or merely a coincidental observation. Moreover, this case emphasizes the potential for misdiagnosis between ALAs and pyogenic liver abscesses, as seen with the patient first being managed for a pyogenic abscess. The absence of typical gastrointestinal symptoms, such as abdominal cramping and diarrhea, which are commonly associated with E. histolytica infection, contributed to the diagnostic challenge in this case. This underscores the importance of increasing awareness regarding the parasite's ability to stay inactive for an extended period before presenting as a liver abscess. Serological tests for E. histolytica could have helped clarify this patient and should be considered, especially for high-risk patients who might not initially exhibit traditional symptoms, for faster diagnosis.

Additionally, we highlight the potential benefit of a CT angiogram for patients with significant liver abscesses. This is to assess the concurrent risk of PE, given that large abscesses might exert pressure on surrounding blood vessels, raising PE risk, a complication that was incidentally detected in our patient without evident DVT. There is limited research on the correlation between PE and amebiasis, but further studies into the association and development of noninvasive rapid tests would aid in faster diagnosis and better patient outcomes.
